# Embarking on a memory assessment voices of older persons living with memory impairment

**DOI:** 10.1177/1471301220910637

**Published:** 2020-03-18

**Authors:** Marie Tyrrell, Dorota Religa, Bjöörn Fossum, Ragnhild Hedman, Kirsti Skovdahl, Pernilla Hillerås

**Affiliations:** Sophiahemmet University, Sweden; NVS, Karolinska Institutet, Sweden; NVS, Karolinska Institutet, Sweden; Sophiahemmet University, Sweden; SöS, Karolinska Institutet, Sweden; Sophiahemmet University, Sweden; Ersta Sköndal Bräcke University College, Sweden; University in South-Eastern Norway, Norway; Sophiahemmet University, Sweden; NVS, Karolinska Institutet, Sweden; Red Cross University College, Sweden

**Keywords:** memory impairment, neuropsychiatric symptoms, primary care, experiences, interviews

## Abstract

**Aim:**

To describe older persons who had commenced a memory assessment, experiences of living with memory impairment and related symptoms.

**Background:**

Persons with subjective memory impairment are two times more likely to develop dementia over the years than their peers. Older persons seldom seek help from primary health care clinics solely for subjective memory impairment. Of those who seek help, it can take up to 35 months from the person experiencing initial symptoms to referral to a memory clinic. Further research is needed regarding how older persons live with memory impairment with related symptoms before they receive a memory diagnosis.

**Method:**

A qualitative study with 23 participants who had commenced a memory assessment in primary care. Semi-structured interviews were held. During the interviews, the Neuropsychiatric Inventory was completed and discussed with the participants. Interview data were analysed using Interpretive Description.

**Results:**

The results are presented under four themes: Conflicting views about the situation, Unveiling the presence of neuropsychiatric symptoms, Compensating with external and internal strategies to recall and Worrying about self and future. Persons with memory impairment were encouraged by family members or others to seek a memory assessment. Few persons were self-referred as memory impairment was often seen as a part of aging. Polarised viewpoints existed within the families regarding the impact of memory impairment on daily life. The presence of neuropsychiatric symptoms appeared unexplored in the participants seeking a memory assessment. In this study, the majority of participants experienced neuropsychiatric symptoms at the time of contact for a memory assessment.

**Conclusions:**

Memory problems experienced were often viewed by the person as being part of an aging process. The presence of neuropsychiatric symptoms was not acknowledged as being connected to memory impairment. Contextualising ‘memory impairment’ as a part of a ‘cognitive process’ may help the person in identifying the presence of neuropsychiatric symptoms.

## Background

Dementia is a global public health priority with an estimated 47 million persons living with the syndrome, the number is said to triple by the year 2050. Globally, dementia is ‘under-detected, under-diagnosed, under-disclosed, under-treated and under-managed in primary care (Prince et al., 2016, p. 7).

Alzheimer’s disease [AD] and other dementias are regarded as a major health problem for persons 65 years and older (Hebert et al., 2013). Wilson et al. (2012) refer to the presence of cognitive impairment in older persons which can exist up to 7.5 years prior to the onset of dementia. During the course of AD changes in the person’s self-awareness of memory function occurs (Vannini et al., 2017). According to Roberts and Knopman (2013), an estimated 15% to 20% of persons over 65 years live with mild cognitive impairment and approximately one third progress to develop dementia. Mitchell et al. (2014) state that persons with subjective memory impairment are two times more likely to develop dementia over the years than their peers. In Sweden, many older persons reside independently in their own homes (Wimo et al., 2017) this includes persons with dementia, with up to 50% who live alone (Cermakova et al., 2017).

The majority of persons during the dementia trajectory experience neuropsychiatric symptoms [NPS] (Lyketosa et al., 2011). Symptoms referred to are, for example, delusions, aggression, anxiety and apathy (Cummings et al., 1994). In longitudinal studies following persons over the dementia disease trajectory, NPS were present at baseline increasing (Vik-Mo et al., 2018) significantly and were consistent during the course of the disease (Brodaty et al., 2015). Hill et al. (2018) identified that persons with mild cognitive impairment commonly experience affective symptoms like depression and anxiety.

A timely diagnosis can facilitate advance care planning (National Institute for Health and Care Excellence, 2018), this includes access to support, services, prescribing potential medications and providing information (Porok et al., 2013). Furthermore, a timely diagnosis can help avoid financial exploitation (Manthorpe et al., 2012) and reduce caregiver fatigue (Park et al., 2015) and is regarded as cost effective for society (Prince et al., 2011). The Swedish National Guidelines for Care of Persons with Dementia recommend that persons with suspected memory impairment should be offered a memory assessment within primary health care (National Board of Health and Welfare, 2017). Primary health care is often the first line of contact for persons or families of persons with concerns regarding memory impairment or cognitive decline (Bunn et al., 2012; Galvin & Sadowsky, 2012).

Older persons seldom seek help solely for subjective memory impairment, limited research is available regarding how older persons view their memory difficulties and how they seek assistance (Begum et al., 2012). Persons can view their situation as a possible threat to their autonomy, sense of security and that it affects their meaningful role in society; however, this depends on the person concerned (Steeman et al., 2006). Campbell et al. (2016) studied older persons who sought assistance for memory impairment and described a transition process involving; onset, identifying symptoms, seeking assistance and diagnosis. Daily life for the persons concerned was overshadowed by a sense of uncertainty. It can take an average of 35 months from the person experiencing initial symptoms to referral from primary health care to a memory assessment clinic (Koskas et al., 2018). Lafortune et al. (2015) highlighted a fragmented primary health care service which can create barriers for the older adult to access services. Perry-Young et al. (2018) identified in their study that ‘discounting, misattributing and deferring’ of memory impairment were some of the reasons for persons in delaying seeking contact with health care for memory issues.

Further knowledge is required surrounding older persons experience of living with memory impairment and their needs in a community setting prior to receiving a dementia diagnosis (Parsons et al., 2013). This study was undertaken with a view to expand the knowledge by capturing the experiences of older persons with memory impairment who had commenced a memory assessment.

## Aim

To describe older persons who had commenced a memory assessment, experiences of living with memory impairment and related symptoms.

## Method

This is a qualitative interview study which aims to gain insights and understandings of a phenomenon from the persons’ who are being interviewed point of view (Kvale & Brinkmann, 2015). In this study, the person’s experiences of living with memory impairment is in focus. An interpretative description [ID] approach as described by Thorne (2016) was used to analyse the interview data in this study. ID is a means of designing a study encompassing the knowledge and conceptual base of the health care discipline of the researcher.

The purpose of ID is threefold: to answer a real-world question, identifying empirical knowledge of the field and awareness of the contextual and conceptual ‘realm’ of the audience. It is a means of engaging with identifiable questions from the applied disciplines of health care departing from ‘self-evident’ knowledge to a more interpretative level of knowledge. It builds on both clinical and evidence-based knowledge and creates an opportunity to identify new insights. These new insights can help shape new practices and inquiries (Thorne, 2016).

This study presents initial interview data of a repeat interview qualitative research study. Interviews were carried out with 23 study participants at their basic memory assessment prior to memory diagnosis. Interviews were held during October 2017 to December 2018.

### Sample

A purposeful sampling strategy was used to recruit participants who through their experiences could greatly enhance the study with their contributions (Polit & Beck, 2017). Inclusion criterion for this study were persons over 65 years residing in a community setting, with a Mini Mental State Examination [MMSE] (Folstein et al., 1975) scores over 15 points and who had commenced a memory assessment in primary health care. A total of 31 persons were invited by health care personal at a primary health care clinic to join the study. Eight persons later declined participation in the study mainly due to time restraints. When interviewing began, 23 persons were interested in participation and included in the study (Table 1). Upon receiving confirmation from the primary health care clinic that the participants wished to join the study, contact was taken by the first author (MT) per telephone.

**Table 1.** Study participants demographics, MMSE scores and NPS self-reported.

Gender/Age of study participant	Living arrangements	MMSE score	NPS self-reported
#1 Male 87 years	Living alone	21/30	Hallucinations
#2 Male 85 years	Living with partner	16/30	Aberrant motor behaviour
#3 Male 85 years	Living with partner	24/30	Irritability
#4 Male 86 years	Living with partner	27/30	Night time behaviour, Appetite
#5 Male 76 years	Living alone	28/30	Depression, Apathy, Irritability, Appetite
#6 Male 91 years	Living with partner	20/30	None
#7 Female 91 years	Living alone	28/30	None
#8 Male 80 years	Living with partner	29/30	Night time behaviour, Anxiety
#9 Female 82 years	Living alone	28/30	Night time behaviour, Anxiety
#10 Female 76 years	Living with partner	28/30	None
#11 Male 82 years	Living with partner	24/30	Irritability, Depression
#12 Female 76 years	Living alone	29/30	None
#13 Male 80 years	Living with partner	28/30	Agitation, Appetite
#14 Female 83 years	Living alone	27/30	Appetite
#15 Male 81 years	Living with partner	29/30	Appetite, Depression
#16 Male 70 years	Living with partner	30/30	Agitation, Depression, Apathy, Irritability
#17 Male 78 years	Living with partner	29/30	Night time behaviour, Irritability, Depression
#18 Male 79 years	Living with partner	30/30	Agitation, Apathy
#19 Male 88 years	Living with partner	29/30	None
#20 Female 79 years	Living alone	28/30	Night time behaviour
#21 Male 73 years	Living with partner	30/30	Agitation
#22 Male 90 years	Living alone	27/30	None
#23 Male 78 years	Living with partner	27/30	None

MMSE: Mini Mental State Examination; NPS: neuropsychiatric symptoms.

### Data collection

Each participant was contacted by the first author to arrange a convenient time and place for the interview. Interviews were held as per participant’s request; in their home, in the first author’s place of work and in two cases, where the participants were self-employed, in their place of employment.

An interview guide with semi-structured questions was used in this study. Semi-structured questions provide a means for the researcher to cover the topic of interest and allow participants to talk freely about the topic under discussion (Polit & Beck, 2017). Questions included were; what it is like to live with memory impairment, and how does memory impairment affect your life. A question regarding if the participant experienced other symptoms which they thought could be related to memory impairment was posed. The Neuropsychiatric Inventory [NPI] (Cummings et al., 1994) was then presented and completed with the participant. The NPI presents a list of 12 NPS. The 12 symptoms listed in the NPI are hallucinations, delusions, depression, apathy, anxiety, irritability, agitation/aggression, euphoria, disinhibition, aberrant motor behaviour, night time behaviour and appetite. Each NPS in the NPI was discussed with the participants, and upon recognition of the presence of an NPS, it was important to establish that the symptom/s were newly debuted. The Neuropsychiatric Inventory Nursing Home [NPI-NH] version (Cummings et al., 1994) was the NPI instrument of choice in this study. To the authors’ knowledge, the only available NPI instrument, at the time of the study, translated and validated in Swedish. Terms pertaining to a nursing home context in the NPI-NH were excluded in the interview. The NPI-NH facilitates the possibility to report frequency, severity and distress related to the NPS for the person concerned. Questions regarding frequency, severity and distress were posed in the interview when it was deemed relevant for the study participant.

Thorne (2016) states how ‘counting’ and observing prevalence is a common way to interpret the world and although the use of numbers is not the mainstay of qualitative research, it can however enhance the transformation of data to results. ID as an approach facilitates the use of various methods of data collection as long as they are aligned with the research focus and the ‘disciplinary knowledge objectives’ (Thorne, 2016). In this study, the inclusion of the NPI helped the study participants to identify the possible presence of NPS (Table 1).

The duration of each interview ranged from 30 to 75 minutes. Upon receiving permission from the participants, 19 of the interviews were audio-recorded, on a code lock audio device, by the first author. Notes were taken during all interviews, and the interviews were transcribed by the first author, directly after each interview.

### Data analysis

Using an ID approach, the data analysis process was initiated and led by the first author who read through the transcribed interviews several times, and relationships between texts were identified forming patterns. Thorne (2016) describes the importance of testing relationships among data to assist the emerging analysis. The first author then clustered the patterns which developed and formed common themes (Table 2). Academic discussions, regarding initial patterns and themes, were held with the research team. Thorne (2016) warns about the risk of over-estimating patterns and the importance of the researcher to hold a critical approach to frequency of topics in the text. In attempts to avoid ‘premature closure’ (Thorne, 2016, p. 194), initial patterns and themes were reassessed and discussed by the research team several times prior to agreement on final patterns and themes.

**Table 2.** Examples of patterns from the data analysis presented as themes.

Patterns	Themes
- Mainly an issue for family - Memory decline a part of aging	Conflicting views about the situation
- Reminding others of situation - Use of technology	Compensating with external and internal strategies to recall
- Neuropsychiatric symptoms	Unveiling the presence of neuropsychiatric symptoms
- Self and identity threatened - Social withdrawal	Worrying about self and future

### Ethical considerations

This study was approved by the Regional Ethical Review Board. All participants received written and verbal information about the study during the recruitment process. Participants were informed that their participation was voluntary and were reminded that they could at any point of time opt to withdraw from the study without consequences. Informed consent was gained from the participants in accordance with the Declaration of Helsinki (World Medical Association Declaration of Helsinki, 2013) which includes that the rights and interests of the study participants must take precedence over the goal of the research.

## Results

The results are presented under four themes: Conflicting views about the situation, Unveiling the presence of NPS, Compensating with external and internal strategies to recall and Worrying about self and future.

### Conflicting views about the situation

Study participants described their own perspective of living with memory impairment and that of others (family, peers and physicians). Participants highlighted how polarised standpoints existed in their families regarding the presence and impact of memory impairment. For participants, concerns about memory impairment had faded in significance in comparison to other aspects of life and general well-being. Family members were portrayed as having contrasting views focusing on the impact memory impairment had on relationships and daily life.

Study participants viewed their memory impairment as part of an aging process which was toned down as having little to no impact on their life. Memory impairment was rationalised as the result of wear and tear on the body due to biological aging.

I have started to understand that I need to get used to living like this, prepare myself for forgetting things, in a way I think when you have turned 81 and have worked your entire bloody life, it’s ok to be forgetful.

A sense of frustration regarding the ‘ownership’ of memory impairment as a problem was present. Participants presented their situation more as a family problem as opposed to a problem for themselves. There was an awareness that being forgetful had become a source of annoyance for family members resulting in aggravating conversations. The participants lived with constant reminders from partners’ or adult children to recall aspects of life which they would likely forget.

My partner is constantly nagging me that I have forgotten things, I know I forget things … it’s my partner that is mostly affected, she says don’t tell me you have forgotten it … no you cannot have forgotten it, and of course I have [embarrassing laugh]

Participants experienced a mismatch of expectations of recall between themselves and their families. Selective attention in conversations and situations was used by participants to recall. By concentrating on specific aspects of a conversation or situation, as opposed to trying to focus on an entire encounter, this facilitated the likely hood that they would recall the encounter. Family members on the other hand were depicted as lacking understanding with expectations of an entire recall of the encounter. Family were portrayed as overly concerned about memory impairment and the impact it had.

Over the last six months my wife has been telling me that I have forgotten this and have forgotten that, but I can’t remember everything she does, I mean we are two different people what I deem important, I can recall.

For some, memory impairment was not recognised or was dismissed by friends and peers as inconsequential. Memory impairment was seen more as a subjective observation which was often not confirmed by others, this was a cause of irritation for the participants. In other situations, participants were confronted by friends and peers about seeking help for their memory impairment. Friends were allies whom they met on equal terms and their opinions were valued.

I met some old work-mates … we talked away and then I forgot what we were discussing, so they said to me … you should get your memory checked out … I mean it can be so bad that you have actually developed memory loss.

Few participants contacted their primary health care clinic solely for memory issues. Memory assessments were instead proposed by others, most commonly partners, who played a pivotal role in ensuring contact was taken. Pleasing partners by actively doing something about the perceived memory impairment was an important factor to avoid further disputes in the home. Overall, participants did not experience their memory impairment as a significant health issue which warranted medical attention or investigation, it was instead problematised and medicalised by others. Despite risks of medicalising what was regarded as a normal situation, participants embarked on a memory assessment without protest.

First of all, it wasn’t me who wanted this [memory assessment], they contacted me. Out of the blue I got a letter from them [primary health care], I thought it was ok and accepted the offer …

Experiences of contact with primary health care for subjective memory impairment concerns did not always meet expectations. A couple of participants, regarded as having high cognitive function, had visited their GPs regarding concerns over memory. The battery of testing offered for memory impairment in primary health care were experienced as elementary and degrading, failing to detect or meet the participants levels of cognitive or memory impairment.

I was told that my [memory test] results were very good. They didn’t think that I needed to go any further … This surprised me somewhat as I would say it was a harmless memory assessment … can you fold this piece of paper in the centre and so … [laugh].

### Unveiling the presence of neuropsychiatric symptoms

Exploring if study participants had developed other symptoms parallel to developing memory impairment was not an easy task. Participants had great difficulties in spontaneously identifying potential associated symptoms. Memory impairment was the ‘mother symptom’ with consequences such as forgetfulness and disorientation viewed as sub-symptoms. Of those participants who had developed physical symptoms, such as headaches and back pain, these symptoms were tangible and easier to identify and discuss.

When discussing the possible presence of NPS associated with memory impairment, study participants appeared both perplexed and curious. Perplexed, surrounding the possibility that memory impairment could be associated with other symptoms, which were latent and could now be revealed and were not part of the aging process. At the same time, a sense of curiosity and eagerness existed to hear about potential symptoms which were unexplored and undetected. When the list of NPS from the NPI was presented, recognition and confirmation of certain symptoms, which had not been discussed earlier, was shared by the majority of the participants despite variations in MMSE scores.

I don’t think that I have any other symptoms … [read NPI list] I think I have become more aggressive, if that has something to do with memory problems I don’t know … I take very little initiative [these days] … it is often my wife who is the driving force, I would prefer to stay at home.

The NPI (Cummings et al., 1994) assisted the participants in identifying the presence of NPS, often for the first time, and appeared to help contextualise the symptoms experienced. The majority of the participants reported the presence of one to two NPS, and two participants reported the co-existence of four NPS. Symptoms such as irritability, depression, sleep disturbances and reduced appetite were most commonly reported (Table 1).

Mentioning certain symptoms listed in the NPI (Cummings et al., 1994) such as hallucinations, disinhibition, euphoria and delusions appeared amusing for some participants who responded with outbursts of laughter and humorous comments. For them, the initial thoughts of developing such symptoms were absurd and beyond belief as they represented a loss of self-control and ability to regulate.

I don’t experience delusions [laugh] however I have problems with others who have. e.g. like politicians … I don’t have depression instead I have a straight line which I would like to think that one day would curve upwards to a bit of euphoria … I only experience euphoria when I go to the theatre.

### Compensating with external and internal strategies to recall

Managing daily life alongside expectations from self and others in the face of memory impairment required strategies. Strategies were created and adapted by participants to maximise performance and compensate for memory impairment. Internal and external strategies were described. Internal strategies involved using themselves as a tool and external strategies involved the use of physical memory tools.

Documentation was a common external strategy used. To document upcoming events, appointments, etc. in an accessible diary or a calendar had become an essential part of life and helped maintain a sense of structure and normality. Technology was another external strategy commonly used, mobile phones were mentioned as valuable sources of help to keep track of time and recall words or events.

I try to systemise my life, but it doesn’t always work [laugh] … I carry a diary in which I have all sorts of things documented [shows and opens the diary] **this is my life** … if it’s not documented that I should pick my grandchildren up then I don’t.

Family involvement in daily life was often appreciated. Family members were seen as facilitators in some cases, offering support and helping to fill in gaps, when required.

I can’t say that I need to apologise for myself, I have accepted what has happened. I have my son, he is my external memory … If it continues like this, then I am ok with it.

Several compensatory internal strategies were shared by the participants. To minimalise details surrounding a situation eliminating unnecessary information was a common means to recall an event. Other strategies to recall words, which were missing or obstructed in conversations, were explained. They involved making word associations or rhythms. These were often silent strategies, time consuming and demanded patience.

… emmm my wife has a plant by the window, to remember the name of the plant, I need to go on a detour with words and think, if I am able to [‘orkar’] water the plant, I get ‘orkar’, then I can retrieve the name orchid [‘orkidéér’]. A direct connection to the word doesn’t exist for me

Forgetting names of friends and peers was regarded as challenging and embarrassing for participants. In attempts to avoid offending persons involved, it was important not to reveal that the person’s name had not come to mind. It was crucial to assess the situation, await cues from others gathered or substitute the person’s name with an endearing term. For other participants, it was necessary to remind people in their company, that they had difficulties in remembering.

It’s like this, I have to remind people around me that I forget!

### Worrying about self and future

Memory impairment entailed losses and feelings of vulnerability in the face of an uncertain future. The term ‘memory impairment’ was a synonym for cognitive impairment. It was not an isolated cognitive symptom, it came hand in hand with impairments of thought processing and intellectual functions. Thoughts and worries regarding a progressive cognitive decline impacted on how participants viewed themselves and what the future held.

Deficits in a cognitive domain such as memory was observed by peers and threatened their sense of self and identity impacting on personal and social relationships. This resulted in participants feeling less relied upon and trusted by family and peers. This was a cause of concern and worry, as trust and reliability were attributes which had been valued and were symbolic of them as a person.

[my wife] now regards me as unreliable when she asks me to do things.

Insights were shared by the participants about lacking ability to live up to pre-conceived expectations of friends and peers, whom they had known greater parts of their life. This was experienced in conversations where exchanges of information about shared memories and events were common. There was a reduced desire to participate in such social gatherings.

My former workmates have received information that I have received a ‘blow’ and that I’m the same person I was before, I think that I was good fun back then, I’m not so today … my girlfriend thinks that I can hold a good conversation.

Beliefs that developing memory impairment was part of normal aging were expressed. Getting old was an inevitable stage of life which the participants were reluctantly entering. A future with memory impairment meant a reassessment and modification of what was feasible and worthwhile in life. To slow down and search for meaningful activities that were compatible to their situation was proposed as a possible solution.

Getting involved in politics again is not on the agenda due to my memory problems … I am still president of our resident’s association.

Of the participants who had a family history of dementia, memory impairment was seen as a potential prelude to dementia. Fears of developing dementia were both implied as well as expressed in the interviews.

I don’t want this to get any worse so that I end up disappearing into the mist … that’s why I need to suss out in advance what’s going on up there in the brain department!

## Discussion

The main findings in this study highlight how participants with memory impairment viewed their situation as a part of an aging process. This is confirmed by Beard and Neary (2013) who described how older persons did not always view memory problems as a pathological development. Participants in this study explained how their memory impairment was problematised by family members placing strains on family life. Most of the participants were encouraged by others to seek a memory assessment, and few participants were self-referred to their primary health care. This is in keeping with a study carried out by Perry-Young et al. (2018) who identified that ‘discounting, misattributing and deferring’ of memory impairment were some of the reasons for persons in delaying seeking contact with health care for memory issues (p. 11). Fears of stigma and social isolation (Phillipson et al., 2015) are other reasons why persons refrain from contacting health care for subjective memory impairment. Furthermore, lack of insight and initiative taking can be viewed as early symptoms in dementia. These symptoms are included in the cognitive domains; social cognition and executive function, which can be affected in both mild and major neurocognitive disorder according to the Diagnostic and Statistical Manual of Mental Disorders fifth edition [DSM-5] (American Psychiatric Association, 2013). Self-awareness of cognitive impairment varies in the early stages of AD according to Hill et al. (2016). Clare (2003) in a seminal study identified how persons with AD processed self-awareness of perceived difficulties related to their disease. Processes were divided up into two domains: self-maintaining and self-adjusting. In this study, participants described how they compensated for memory impairment, normalising and minimalising impact on their lives prior to memory diagnosis which according to Clare (2003) are themes seen in self-maintaining.

Further findings in this study highlight that participants seeking a memory assessment were unaware of the presence of NPS. Prior to the introduction of NPI, participants had difficulties in identifying possible co-related symptoms to memory impairment. The majority of participants identified, with the help of the NPI (Cummings et al., 1994), that they experienced one or more NPS. NPS identified were new symptoms which had gradually developed over time and were not overtly associated with memory impairment. It was often the first time the participants had identified and spoken about these symptoms. To the authors’ knowledge, the NPI is not used in primary care or in specialist memory clinics for persons seeking memory assessments. Borsje et al. (2019) describe how community residing persons with dementia are living with high levels of NPS. According to Karttunen et al. (2011), approximately 80% of persons, in the very early stages of AD, reported the presence of NPS. Borsje et al. (2019) highlight the need for primary care to investigate the presence and follow-up NPS in persons with dementia. Hill et al. (2018) described the impact memory impairment had on persons with mild cognitive impairment, it varied from frustration to social withdrawal depending on the meaning placed on the experienced deficits. One participant in this study reluctantly reported the presence of hallucinations which was not openly discussed before, and other participants reported NPS such as agitation/aggression and irritability which appeared directly related to frustration surrounding memory impairment. Loss of appetite was reported by some participants. The Swedish Dementia Registry annual report (SveDem, 2017) identified that one third of persons with dementia are undernourished with a body mass index of under 22. Examples of other NPS reported in this study were depression and anxiety. Hill et al. (2016) describe how subjective memory impairment is associated with depressive and anxiety symptoms.

Keady and Jones (2010) described, in their case study, the significance of using a person-centred approach in understanding why a person with dementia exhibited NPS in a nursing care facility. This study shows how older persons prior to a cognitive diagnosis were living with NPS in a community setting. The use of the NPI helped highlight the presence of NPS which otherwise could go undetected during the memory assessment. Using a person-centred approach in primary care and completion of the NPI can assist in the identification of NPS and provision of follow-up care and support.

In this study, the term memory impairment was not contextualised, for the participant, as one of several cognitive processes influencing behaviour and emotions. Such explanation may have helped the participant identify previously undetected cognitive symptoms and NPS. In an international multi-centre study, Rabin et al. (2015) identified that memory was the most common cognitive domain investigated in older persons experiencing subjective cognitive decline. Reisberg and Gauthier (2008) discuss how subjective cognitive impairment is commonly known as subjective memory complaint. However, they recommend the use of the term cognitive decline as opposed to the term memory impairment due to the complexity of the field and by saying cognitive decline it is not limited to memory alone.

According to Morgan et al. (2014) family members encouraged contact with a memory clinic to gain validation about symptoms experienced and to receive a diagnosis for future planning. In the event the person received a dementia diagnosis, it facilitated an understanding of their situation; furthermore, a diagnosis helped the person with memory impairment to accept their situation (Morgan et al., 2014). One participant explained how he received an invite to commence a memory assessment, the participant speculated that it was part of a routine health check and accepted the offer. To systematically offer memory assessments to persons over a certain age may influence the number of referrals and help identify persons at risk of developing dementia or cognitive impairment.

Receiving an early dementia diagnosis can assist the person in planning future care with focus on interventions and support (National Institute for Health and Care Excellence, 2018; Porok et al., 2013). However, treating physicians are sometimes reluctant in setting a dementia diagnosis due to concerns of the potential stigmatisation (Gove et al., 2016) and in the absence of available interventions and social support (Smith et al., 2017). This may further delay the process of diagnosing dementia for the persons concerned. It is a process for the person themselves to recognise if symptoms experienced warrant a medical investigation or not. According to Steeman et al. (2006) in the pre-diagnostic phase, persons can fluctuate between both awareness and denial which can exist simultaneously. Not all of the participants in this study were eligible to continue a memory assessment after the initial visit to their primary health care. The participants concerned scored full marks on the basic cognitive screening, and during the initial assessment, the treating physician evaluated that there was no need to proceed with a memory assessment. These two participants were high-functioning individuals and were surprised how standardised and basic the testing was. Both study participants reported the presence of NPS when completing the NPI. According to National Institute for Health and Clinical Excellence (2018), the risk of developing dementia can exist in persons with normal scores in cognitive testing. Not confirming the person’s worries about subjective cognitive concerns leaves the situation unresolved and the person is left in an ambiguous position (Birt et al., 2019). Cognitive testing offered in primary health care includes the completion of the MMSE (Folstein et al., 1975), which according to Breton et al. (2019), has lower sensitivity than other cognitive screening instruments.

Limitations in this study were that participants were recruited from one primary health care clinic in a city in Sweden. No data were gathered about the participants’ level of education; despite this, many of the participants appeared to have university-level education.

## Conclusion

Memory problems experienced were often viewed by the person as being part of an aging process. The presence of NPS was not acknowledged as being connected to memory impairment. Contextualising ‘memory impairment’ as a part of a ‘cognitive process’ may help the person in identifying the presence of NPS.

## Implications for practice

Older persons may view their memory impairment as part of an aging process and therefore not see the need to seek a memory assessment. Primary care should be aware that screening for NPS as part of a memory assessment can help identify the presence of symptoms which otherwise can remain undetected and unmanaged for the person concerned.

## References

[bibr1-1471301220910637] American Psychiatric Association. (2013). *Diagnostic and statistical manual of mental disorders: DSM-5* (5th ed.).

[bibr2-1471301220910637] Beard L. R. Neary T. M. (2013). Making sense of nonsense: Experiences of mild cognitive impairment. Sociology of Health & Illness, 35(1), 130–146. 22554111 10.1111/j.1467-9566.2012.01481.x

[bibr3-1471301220910637] Begum A. Morgan C. Chiu C. C. Tylee A. Stewart R. (2012). Subjective memory impairment in older adults: Aetiology, salience and help seeking. International Journal of Geriatric Psychiatry, 27(6), 612–620. 21766337 10.1002/gps.2760

[bibr4-1471301220910637] Birt L. Poland F. Charlesworth G. Leung P. Higgs P. (2019). Relational experiences of people seeking help and assessment for subjective cognitive concern and memory loss. Aging & Mental Health, 27, 1–9. 10.1080/13607863.2019.1592111 30917667

[bibr5-1471301220910637] Borsje P. Lucassen P. L. B. J. Bor H. Wetzels R. B. Pot A. M. Koopmans R. T. C. M. (2019). The course of neuropsychiatric symptoms in patients with dementia in primary care. Family Practice, 36(4), 437–444. 30517631 10.1093/fampra/cmy117

[bibr6-1471301220910637] Breton A. Casey D. Arnaoutoglou N. A. (2019). Cognitive tests for the detection of mild cognitive impairment (MCI), the prodromal stage of dementia: Meta‐analysis of diagnostic accuracy studies. Int J Geriatr Psychiatry, 34(2), 233–242. 30370616 10.1002/gps.5016

[bibr7-1471301220910637] Brodaty H. Connors M. H. Xu J. Woodward M. Ames D., & PRIME Study Group. (2015). The course of neuropsychiatric symptoms in dementia: A 3-year longitudinal study. Journal of the American Medical Directors Association, 16(5), 380–387. 25687925 10.1016/j.jamda.2014.12.018

[bibr8-1471301220910637] Bunn F. Goodman C. Sworn K. Rait G. Brayne C. Robinson L. McNeilly E. Iliffe S. (2012). Psychosocial factors that shape patient and carer experiences of dementia diagnosis and treatment: A systematic review of qualitative studies. PLoS Medicine, 9(10), e1001331. 23118618 10.1371/journal.pmed.1001331 PMC3484131

[bibr9-1471301220910637] Campbell C. Manthorpe J. Samsi K. Abley C. Robinson L. Watts S. Bond J. Keady J. (2016). Living with uncertainty: Mapping the transition from pre-diagnosis to a diagnosis of dementia. Journal of Aging Studies, 37, 40–47. 27131277 10.1016/j.jaging.2016.03.001

[bibr10-1471301220910637] Cermakova P. Nelson M. Secnik J. Garcia-Ptacek S. Johnell K. Fastbom J. Kilander L. Winblad B. Eriksdotter M. Relgia D. (2017). Living alone with Alzheimer’s disease: Data from SveDem, the Swedish Dementia Registry. Journal of Alzheimer’s Disease, 58(4), 1265–1272. 10.3233/JAD-170102 PMC5523910 28550260

[bibr12-1471301220910637] Clare L. (2003). Managing treats to self: Awareness in early stage Alzheimer’s disease. Social Science & Medicine, 57, 1017–1029. 12878102 10.1016/s0277-9536(02)00476-8

[bibr13-1471301220910637] Cummings J. L. Mega M. Gray K. Rosenberg-Thompson S. Carusi D. A. Gornbein J. (1994). The Neuropsychiatric Inventory: Comprehensive assessment of psychopathology in dementia. Neurology, 44(12), 2308–2314. 7991117 10.1212/wnl.44.12.2308

[bibr14-1471301220910637] Folstein M. Folstein S. E. McHugh P. (1975). Mini-mental state: A practical method for grading the cognitive state of patients for the clinician. Journal of Psychiatric Research, 12, 189–198. 1202204 10.1016/0022-3956(75)90026-6

[bibr15-1471301220910637] Galvin J. E. Sadowsky C. H. (2012). Practical guidelines for the recognition and diagnosis of dementia. The Journal of the American Board of Family Medicine, 25(3), 367–382. 22570400 10.3122/jabfm.2012.03.100181

[bibr16-1471301220910637] Gove D. Downs M. Vernooij-Dassen M. Small N. (2016). Stigma and GPs’ perceptions of dementia. Aging & Mental Health, 20(4), 391–400. 25765096 10.1080/13607863.2015.1015962

[bibr17-1471301220910637] Hebert L. E. Weuve J. Scherr P. A. Evans D. A. (2013). Alzheimer disease in the United States (2010–2050) estimated using the 2010 census. Neurology, 80(19), 1778–1783. 23390181 10.1212/WNL.0b013e31828726f5 PMC3719424

[bibr18-1471301220910637] Hill J. Mogle J. Kitko L. Gilmore-Bykovskyib A. Wion R. Kitt-Lewis E. Kolanowski A. (2018). Incongruence of subjective memory impairment ratings and the experience of memory problems in older adults without dementia: A mixed methods study. Aging & Mental Health, 22(8), 972–979. 28604058 10.1080/13607863.2017.1337715

[bibr19-1471301220910637] Hill N. L. Mogle J. Wion R. Munoz E. DePasquale N. Yevchak A. M. Parisi J. M. (2016). Subjective cognitive impairment and affective symptoms: A systematic review. The Gerontologist, 56, e109–e127. 27342440 10.1093/geront/gnw091 PMC5181393

[bibr20-1471301220910637] Karttunen K. Karppi P. Hiltunen A. Vanhanen M. Välimäki T. Martikainen J. Valtonen H. Sivenius J. Soininen H. Hartikainen S. Suhonen J. Pirttilä T.; ALSOVA Study Group. (2011). Neuropsychiatric symptoms and quality of life in patients with very mild and mild Alzheimer’s disease. International Journal of Geriatric Psychiatry, 26(5), 473–482. 21445998 10.1002/gps.2550

[bibr21-1471301220910637] Keady J. Jones L. (2010). Investigating the causes of behaviours that challenge in people with dementia. Nursing Older People, 22(9), 10.7748/nop2010.11.22.9.25.c8061 21140883

[bibr22-1471301220910637] Koskas P. Pons-Peyneau C. Houenou-Quenum N. Romdhani M. Gasmi M. Galleron S. Drunat O. (2018). Factors influencing time between onset of signs/symptoms and referral for dementia in elderly outpatients. Revue Neurologique, 174(1–2), 36–43. 28595977 10.1016/j.neurol.2017.05.012

[bibr23-1471301220910637] Kvale S. Brinkmann S. (2015). Interviews learning the craft of a qualitative research interviewing. SAGE.

[bibr24-1471301220910637] Lafortune C. Huson K. Santi S. Stolee P. (2015). Community-based primary health care for older adults: A qualitative study of the perceptions of clients, caregivers and health care providers. BMC Geriatrics, 15, 57. 25925552 10.1186/s12877-015-0052-x PMC4434544

[bibr25-1471301220910637] Lyketosa C. G. Carrillob M. C. Ryanc J. M. Khachaturiand A. S. Trzepacze P. Amatniekf C. Cedarbaum J. Brashear R. Miller D. S. (2011). Neuropsychiatric symptoms in Alzheimer’s disease. Alzheimer’s & Dementia, 7(5), 532–539. 10.1016/j.jalz.2011.05.2410 PMC3299979 21889116

[bibr26-1471301220910637] Manthorpe J. Samsi K. Rapaport J. (2012). Responding to the financial abuse of people with dementia: A qualitative study of safeguarding experiences in England. International Psychogeriatrics, 24(9), 1454–1464. 22464777 10.1017/S1041610212000348

[bibr29-1471301220910637] Mitchell A. J. Beaumont H. Ferguson D. Yadegarfar M. Stubbs B. (2014). Risk of dementia and mild cognitive impairment in older people with subjective memory complaints: Meta-analysis. Acta Psychiatrica Scandinavica, 130(6), 439–451. 25219393 10.1111/acps.12336

[bibr30-1471301220910637] Morgan D. G. Walls-Ingram S. Cammer A. O’Connell M. E. Crossley M. Dal Bello-Haas V. Forbes D. Innes A. Kirk A. Stewart N. (2014). Informal caregivers hopes and expectations of a referral to a memory clinic. Social Science & Medicine, 102, 111–118. 24565148 10.1016/j.socscimed.2013.11.023

[bibr31-1471301220910637] National Board of Health and Welfare. (2017). Nationella riktlinjer för vård och omsorg vid demensssjukdom: Stöd för styrning och ledning [National guidelines for care and care in dementia: Support for management and management]. Socialstyrelsen. https://www.socialstyrelsen.se/globalassets/sharepoint-dokument/artikelkatalog/nationella-riktlinjer/2017-12-2.pdf

[bibr32-1471301220910637] National Institute for Health and Clinical Excellence. (2018). *Dementia: Assessment, management and support for people living with dementia and their carers*. NICE guideline [NG97]. Dementia report, UK. https://www.nice.org.uk/guidance/ng97/chapter/Recommendations#diagnosis 30011160

[bibr33-1471301220910637] Park M. Sung M. Kim S. K. Kim S. Lee D. Y. (2015). Multidimensional determinants of family caregiver burden in Alzheimer’s disease. International Psychogeriatrics, 27(8), 1355–1364. 25853717 10.1017/S1041610215000460

[bibr34-1471301220910637] Parsons K. Surprenant A. Tracey A. M. Godwin M. (2013). Community-dwelling older adults with memory loss: Needs assessment. Canadian Family Physician, 59(3), 278–285. 23486801 PMC3596209

[bibr35-1471301220910637] Perry-Young L. Owen G. Kelly S. Owens C. (2018). How people come to recognise a problem and seek medical help for a person showing early signs of dementia: A systematic review and meta-ethnography. Dementia, 17(1), 34–60. 26764265 10.1177/1471301215626889 PMC5758935

[bibr36-1471301220910637] Phillipson L. Magee C. Jones S. Reisa S. Skaldzien E. (2015). Dementia attitudes and help-seeking intentions: An investigation of responses to two scenarios of an experience of the early signs of dementia. Aging & Mental Health, 19(11), 968–977. 25554920 10.1080/13607863.2014.995588

[bibr37-1471301220910637] Polit D. F. Beck C. T. (2017). Nursing research generating and assessing evidence for nursing practice (10th ed.). Wolters Kluwer.

[bibr38-1471301220910637] Porok J. C. Horgan S. Seitz D. P. (2013). Health care experiences of people with dementia and their caregivers: A meta-ethnographic analysis of qualitative studies. CMAJ, 185(14), E669–E680. 24003093 10.1503/cmaj.121795 PMC3787191

[bibr39-1471301220910637] Prince M. Comas-Herrera A. Knapp M. Guerchet M. Karagiannidou M. (2016). World Alzheimer Report 2016. Improving healthcare for people living with dementia coverage, quality and costs now and in the future. Alzheimer’s Disease International.

[bibr40-1471301220910637] Prince P. M. Bryce D. R. Ferri D. C. (2011). World Alzheimer Report, 2011. The benefits of early diagnosis and intervention. Institute of Psychiatry, King’s College.

[bibr41-1471301220910637] Rabin L. A. Smart C. M. Crane P. K. Amariglio R. E. Berman L. M. Boada M. Buckley R. F. Chételat G. Dubois B. Ellis K. A. Gifford K. A. Jefferson A. L. Jessen F. Katz M. J. Lipton R. B. Luck T. Maruff P. Mielke M. M. Molinuevo J. L. Sikkes S. A. (2015). Subjective cognitive decline in older adults: An overview of self-report measures used across 19 international research studies. Journal of Alzheimer’s Disease, 48(Suppl 1), S63–S86. 10.3233/JAD-150154 PMC4617342 26402085

[bibr42-1471301220910637] Reisberg B. Gauthier S. (2008). Current evidence for subjective cognitive impairment (SCI) as the pre-mild cognitive impairment (MCI) stage of subsequently manifest Alzheimer’s disease. International Psychogeriatrics, 20(1), 1–16. 18072981 10.1017/S1041610207006412

[bibr43-1471301220910637] Roberts R. Knopman D. S. (2013). Classification and epidemiology of MCI. Clinics in Geriatric Medicine, 29, 753–772. 24094295 10.1016/j.cger.2013.07.003 PMC3821397

[bibr44-1471301220910637] Smith T. Cross J. Poland F. Clay F. Brookes A. Maidment I. Penhale B. Laidlaw K. Fox C. (2017). Systematic review investigating multi-disciplinary team approaches to screening and early diagnosis of dementia in primary care—What are the positive and negative effects and who should deliver it? Current Alzheimer Research, 15(1), 5–17. 10.2174/1567205014666170908094931 28891442

[bibr45-1471301220910637] Steeman E. de Casterle D. Godderis J. Grypdonck M. (2006). Living with early-stage dementia: A review of qualitative studies. Journal of Advanced Nursing, 54(6), 722–738. 16796664 10.1111/j.1365-2648.2006.03874.x

[bibr46-1471301220910637] Swedish Dementia Registry (SveDem). (2017). *Annual report*. https://www.ucr.uu.se/svedem/om-svedem/arsrapporter

[bibr47-1471301220910637] Thorne S. (2016). Interpretive description, qualitative research for applied practice (2nd ed.). Routledge.

[bibr48-1471301220910637] Vannini P. Amariglio R. Hanseeuw B. Johnson K. A. McLaren D. G. Chhatwal J. Pascual-Leone A. Rentz D. Sperling R. A. (2017). Memory self-awareness in the preclinical and prodromal stages of Alzheimer’s disease. Neuropsychologia, 99, 343–349. 28385579 10.1016/j.neuropsychologia.2017.04.002 PMC5473166

[bibr49-1471301220910637] Vik-Mo A. O. Gill L. M. Ballard C. Aarsland D. (2018). Course of neuropsychiatric symptoms in dementia: 5-year longitudinal study. International Journal of Geriatric Psychiatry, 33(10), 1361–1369. 29979473 10.1002/gps.4933

[bibr50-1471301220910637] Wilson R. S. Segawa E. Boyle P. A. Anagnos S. E. Hizel L. P. Bennett D. A. (2012). The natural history of cognitive decline in Alzheimer’s disease. Psychology and Aging, 27(4), 1008–1017. 22946521 10.1037/a0029857 PMC3534850

[bibr51-1471301220910637] WimoA.ElmstahlS.FratiglioniL.SjölundB. M.SkoldungerA.FagerströmC.BerglundJ.LagergrenM. (2017). Formal and informal care of community living older people: A population-based study for the Swedish National study on Aging and Care. The Journal of Nutrition, Health and Aging, 21(1), 17–34.10.1007/s12603-016-0747-527999845

[bibr52-1471301220910637] World Medical Association Declaration of Helsinki. (2013). https://www.wma.net/policies-post/wma-declaration-of-helsinki-ethical-principles-for-medical-research-involving-human-subjects/ (accessed May 2019).

